# Whole genome-based phylogeny of reptile-associated *Helicobacter* indicates independent niche adaptation followed by diversification in a poikilothermic host

**DOI:** 10.1038/s41598-017-09091-7

**Published:** 2017-08-21

**Authors:** Maarten J. Gilbert, Birgitta Duim, Arjen J. Timmerman, Aldert L. Zomer, Jaap A. Wagenaar

**Affiliations:** 1 Department of Infectious Diseases and Immunology, Faculty of Veterinary Medicine, Utrecht University, Utrecht, The Netherlands; 2 WHO Collaborating Centre for Campylobacter/OIE Reference Laboratory for Campylobacteriosis, Utrecht, The Netherlands; 3 Wageningen Bioveterinary Research, Lelystad, The Netherlands

## Abstract

Reptiles have been shown to host a significant *Helicobacter* diversity. In order to survive, reptile-associated *Helicobacter* lineages need to be adapted to the thermally dynamic environment encountered in a poikilothermic host. The whole genomes of reptile-associated *Helicobacter* lineages can provide insights in *Helicobacter* host adaptation and coevolution. These aspects were explored by comparing the genomes of reptile-, bird-, and mammal-associated *Helicobacter* lineages. Based on average nucleotide identity, all reptile-associated *Helicobacter* lineages in this study could be considered distinct species. A whole genome-based phylogeny showed two distinct clades, one associated with chelonians and one associated with lizards. The phylogeny indicates initial adaptation to an anatomical niche, which is followed by an ancient host jump and subsequent diversification. Furthermore, the ability to grow at low temperatures, which might reflect thermal adaptation to a reptilian host, originated at least twice in *Helicobacter* evolution. A putative tricarballylate catabolism locus was specifically present in *Campylobacter* and *Helicobacter* isolates from reptiles. The phylogeny of reptile-associated *Helicobacter* parallels host association, indicating a high level of host specificity. The high diversity and deep branching within these clades supports long-term coevolution with, and extensive radiation within the respective reptilian host type.

## Introduction

All *Helicobacter* species are associated with vertebrate hosts, in which they usually colonize the mucosa of the gastrointestinal tract and the liver. Although generally believed to occur primarily in birds and mammals^1^, it has been shown that reptiles host a large *Helicobacter* diversity as well^2, 3^. *Helicobacter* occurrence in reptiles ranges from 4.8% to 39.1%, depending on the detection method used^2^. Based on 16S rRNA phylogeny, *Helicobacter* lineages isolated from reptiles formed a distinct cluster, separate from *Helicobacter* species isolated from mammals and birds^2^. This also suggested confined host association, as the lineages were separated in a cluster of isolates originating from lizards and a cluster of isolates originating from chelonians. These lineages represent up to eight putative novel species, based on 16S rRNA homology (93–98%), indicating that *Helicobacter* biodiversity in reptiles can be considered high, compared to the currently known species from related genera *Arcobacter* (three species) and *Campylobacter* (four species)^2^.

In general, whereas mammals and birds are endothermic and have more constant body temperatures (homeothermic), reptiles are ectothermic and largely dependent on external heat sources for their preferred body temperatures, which can show considerable fluctuations (poikilothermic). Consequently, *Helicobacter* species occurring in poikilothermic reptiles have to be adapted to larger temperature ranges and on average lower temperatures than *Helicobacter* species occurring in homeothermic animals.

Indeed, initial genetic and phenotypic characterization of a *Helicobacter* strain obtained from a western hognose snake (*Heterodon nasicus*) showed that this strain was distinct from other *Helicobacter* species in the ability to grow at lower temperatures (25 °C)^4^. This *Helicobacter* strain, for which the name *Helicobacter serpensis* sp. pr. (species proponenda) has been proposed, had an identical 16S rRNA sequence to *Helicobacter* strain 12S02232-10, which was independently isolated from a rhinoceros iguana (*Cyclura cornuta*), and both strains likely belong to the same species^2^.

No association with disease in reptiles is apparent for *Helicobacter*, and *Helicobacter* likely represents a component of the normal reptilian microbiome, although a fatal septicemia in a pancake tortoise (*Malacochersus tornieri*) has been shown associated with an undescribed *Helicobacter* species^5^.

The obvious host dichotomy and 16S rRNA phylogeny suggested long-term divergence and coevolution between *Helicobacter* and its reptilian hosts^2^. However, from this phylogenetic analysis it remained inconclusive whether the reptile-associated *Helicobacter* lineages are more related to enterohepatic or gastric *Helicobacter* species. Indeed, *Helicobacter* phylogeny based on 16S rRNA has been shown discordant with phylogenies based on other sequence data^6^. In this study, the genomes of *Helicobacter* strains from reptiles were characterized and compared to *Helicobacter* strains from birds and mammals to elucidate the factors contributing to adaptation to poikilothermic or homeothermic hosts, and to gain insights in *Helicobacter* phylogeny and the coevolutionary trajectory of *Helicobacter* and the reptilian host.

## Results

### Phylogeny of reptile-associated Helicobacter

**Figure 1:**
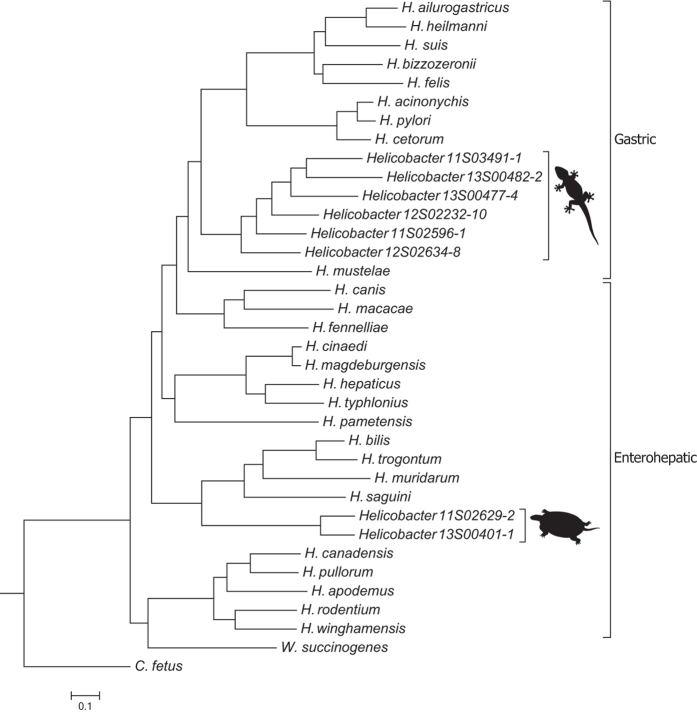
Rooted whole genome-based phylogeny for all *Helicobacter* strains used in this study. The squamate- and chelonian-associated *Helicobacter* clades are indicated with a lizard or chelonian, respectively. *C*. *fetus* strain 82–40 was used as outgroup and root.

A whole genome-based phylogeny accounting for the effects of homologous recombination was reconstructed for the reptile-associated *Helicobacter* strains and (candidate) *Helicobacter* species from birds and mammals (Fig. 1). The most basal split is between the unsheathed *Helicobacter* species, including *Wolinella succinogene*s, and the other enterohepatic and gastric *Helicobacter* species. Notably, *Wolinella succinogenes* does not cluster separately from *Helicobacter*, but forms a separate clade together with the unsheathed *Helicobacter* species. The gastric *Helicobacter* species branch of last from the enterohepatic *Helicobacter* clade. The whole genome-based phylogeny suggests that the gastric *Helicobacter* lineages evolved most recently from an enterohepatic ancestor.

The reptile-associated *Helicobacter* strains form two separate and highly divergent clades, one associated with chelonian hosts and one associated with squamate hosts (i.e. lizards and snakes). These two clades are nested within the *Helicobacter* genus and do not form a basal clade separate from *Helicobacter* species associated with avian and mammalian hosts.

The chelonian-associated *Helicobacter* clade is most closely related to enterohepatic *Helicobacter* species having periplasmic fibers, which wrap helically around the body of the bacterium and give a crisscross appearance to the bacterial surface, a morphologic feature which is often used to subdivide enterohepatic *Helicobacter* species^1^. As many enterohepatic *Helicobacter* species, the chelonian-associated *Helicobacter* strains lack the genes needed for urease production. In contrast, the squamate-associated *Helicobacter* clade is most closely related to gastric *Helicobacter* species. Similar to gastric *Helicobacter* species, the urease locus is conserved in all squamate-associated *Helicobacter* strains. A phylogeny based on urease encoding genes displayed a similar topology as the whole genome-based phylogeny (data not shown).

### Speciation of reptile-associated Helicobacter

The average nucleotide identity (ANI) was calculated to determine whether the different reptile-associated *Helicobacter* lineages can be considered separate species. Supplementary Table S1 shows the ANI for all reptile-associated *Helicobacter* strains and a selection of the most closely related *Helicobacter* species. The ANI largely reflects the phylogeny, as the chelonian- and squamate-associated *Helicobacter* clades are clearly separated. All of the eight reptile-associated *Helicobacter* lineages show an ANI well below the species delimitation of 95–96%, which indicates that each lineage can be considered a novel species based on genetic divergence.

### Thermal adaptation

The growth temperature range was determined for the reptile-associated *Helicobacter* strains and compared to the growth temperature range of the other bird- and mammal-associated *Helicobacter* species (Table 1). Notably, all reptile-associated *Helicobacter* strains, but none of the bird- and mammal-associated *Helicobacter* species, were able to grow at 25 °C. All reptile-associated *Helicobacter* strains showed growth at 37 °C and two of the eight reptile-associated *Helicobacter* strains showed growth at 42 °C.

**Table 1:** Characteristics of the strains used in this study. Characteristics for reference strains are adapted from Schauer^1^, Lawson and Owen^4^, and Haesebrouck *et al*.^25^.

Species	Strain	Host	Host class	Growth temperature			Urease
				25 °C	37 °C	42 °C	
*Helicobacter* 11S02629-2*	11S02629-2	Spur-thighed tortoise (*Testudo graeca*)	Reptilia	+	+	−	−
*Helicobacter* 13S00401-1*	13S00401-1	Central Asian tortoise (*Agrionemys horsfieldii*)	Reptilia	+	+	−	−
*Helicobacter* 11S02596-1*	11S02596-1	Spiny-tailed monitor (*Varanus acanthurus*)	Reptilia	+	+	+	+
*Helicobacter* 11S03491-1*	11S03491-1	Leopard gecko (*Eublepharis macularius*)	Reptilia	+	+	−	+
*Helicobacter* 12S02232-10*	12S02232-10	Rhinoceros iguana (*Cyclura cornuta*)	Reptilia	+	+	−	+
*Helicobacter* 12S02634-8*	12S02634-8	Argentine black and white tegu (*Tupinambis merianae*)	Reptilia	+	+	+	+
*Helicobacter* 13S00477-4*	13S00477-4	Common house gecko (*Hemidactylus frenatus*)	Reptilia	+	+	−	+
*Helicobacter* 13S00482-2*	13S00482-2	Asian grass lizard (*Takydromus sexlineatus*)	Reptilia	+	+	−	+
**Gastric**							
*Helicobacter acinonychis*	Sheeba	Cheetah (*Acinonyx jubatus*)	Mammalia	−	+	−	+
*Helicobacter ailurogastricus*	ASB7	Cat (*Felis catus*)	Mammalia	−	+	−	+
*Helicobacter bizzozeronii*	CIII-1	Human (*Homo sapiens*)	Mammalia	−	+	+	+
*Helicobacter cetorum*	MIT 99-5656	Atlantic white sided dolphin (*Lagenorhynchus acutus*)	Mammalia	−	+	+	+
*Helicobacter felis*	ATCC 49179	Cat (*Felis catus*)	Mammalia	−	+	+	+
*Helicobacter heilmanii*	ASB1.4	Cat (*Felis catus*)	Mammalia	−	+	−	+
*Helicobacter mustelae*	12198	Ferret (*Mustela putorius*)	Mammalia	−	+	+	+
*Helicobacter pylori*	J99	Human (*Homo sapiens*)	Mammalia	−	+	−	+
*Helicobacter suis*	HS1	Pig (*Sus scrofa*)	Mammalia	−	+	−	+
**Enterohepatic (sheated)**							
*Helicobacter bilis*	ATCC 51630	House mouse (*Mus musculus*)	Mammalia	−	+	−	+
*Helicobacter canis*	NCTC 12740	Dog (*Canis lupus*)	Mammalia	−	+	+	−
*Helicobacter cinaedi*	ATCC BAA-847	Human (*Homo sapiens*)	Mammalia	−	+	−	−
*Helicobacter fennelliae*	MRY12-0050	Human (*Homo sapiens*)	Mammalia	−	+	−	−
*Helicobacter hepaticus*	ATCC 51449	House mouse (*Mus musculus*)	Mammalia	−	+	−	+
*Helicobacter macacae*	MIT 99-5501	Rhesus macaque (*Macaca mulatta*)	Mammalia	−	+	−	−
Helicobacter magdeburgensis*	MIT 96-1001	House mouse (*Mus musculus*)	Mammalia	−	+	+	−
*Helicobacter muridarum*	ST1	House mouse (*Mus musculus*)	Mammalia	−	+	−	+
*Helicobacter pametensis*	ATCC 51478	Common tern (*Sterna hirundo*)	Aves	−	+	+	−
*Helicobacter saguini*	MIT 97-6194	Cotton-top tamarins (*Saguinus oedipus*)	Mammalia	−	+	+	−
*Helicobacter trogontum*	ATCC 700114	Norway rat (*Rattus norvegicus*)	Mammalia	−	+	+	+
*Helicobacter typhlonius*	MIT 97-6810	House mouse (*Mus musculus*)	Mammalia	−	+	+	−
**Enterohepatic (unsheated)**							
Helicobacter apodemus*	MIT 03-7007	Korean striped field mouse (*Apodemus agrarius*)	Mammalia	−	+	+	+
*Helicobacter canadensis*	MIT 98-5491	Human (*Homo sapiens*)	Mammalia	−	+	+	−
*Helicobacter pullorum*	MIT 98-5489	Chicken (*Gallus gallus*)	Aves	−	+	+	−
*Helicobacter rodentium*	ATCC 700285	House mouse (*Mus musculus*)	Mammalia	−	+	+	−
Helicobacter winghamensis*	ATCC BAA-430	Human (*Homo sapiens*)	Mammalia	−	+	−	−
*Wolinella succinogenes*	DSM 1740	Bovine (*Bos taurus*)	Mammalia	−	+	+	−

*Candidate *Helicobacter* species.

### Genetic features specific to reptile-associated Helicobacter

The genomes of all reptile-associated *Helicobacter* strains were screened for specific genetic features (Supplementary Table S2). A total of 17 genes, encoding mostly hypothetical proteins and two outer membrane proteins, were specific for the lizard-associated *Helicobacter* clade. Both chelonian-associated *Helicobacter* lineages specifically shared 148 genes. All reptile-associated *Helicobacter* lineages collectively shared three genes, encoding an acetyltransferase (GNAT) family protein, putative methyltransferase YcgJ, and glutamyl-tRNA amidotransferase subunit A. The urease locus was present in all lizard-associated *Helicobacter* lineages, but absent from all chelonian-associated *Helicobacter* lineages. Additional urease alpha and beta subunits were present in the genomes of *H*. *acinonychis*, *H*. *cetorum*, *H*. *felis*, and *H. mustelae*.

The amino acid sequences of each genome were searched for amino acid sequences of known *H*. *pylori* virulence factors (AlpA, AlpB, BabA, CagA, DupA, gGT, HopZ, IceA, IceA2, NapA, OipA, SabA, VacA). Most of these virulence factors (10–12) were also present in the closely related *H*. *acinonychis* and *H*. *cetorum* (Supplementary Table S3). In all other strains, including the reptile-associated *Helicobacter* lineages, few of these virulence factors (2–5) were present. In all reptile-associated *Helicobacter* lineages gGT and NapA were present, HopZ was present only in the lizard-associated *Helicobacter* lineages, and DupA was present in all lizard-associated *Helicobacter* lineages except *Helicobacter* 11S02596-1 and 12S02634-8.

Notably, as in reptile-associated *Campylobacter* lineages, genes of a putative tricarballylate catabolism locus *tcuRABC* were present in all reptile-associated *Helicobacter* lineages. Outside the reptile-associated *Helicobacter* lineages, these genes were only observed in the distantly related species *H*. *canadensis* and *H*. *macacae*. As the *tcuRABC* locus was incomplete in most lineages, the functionality may be altered or impaired.

**Figure 2:**
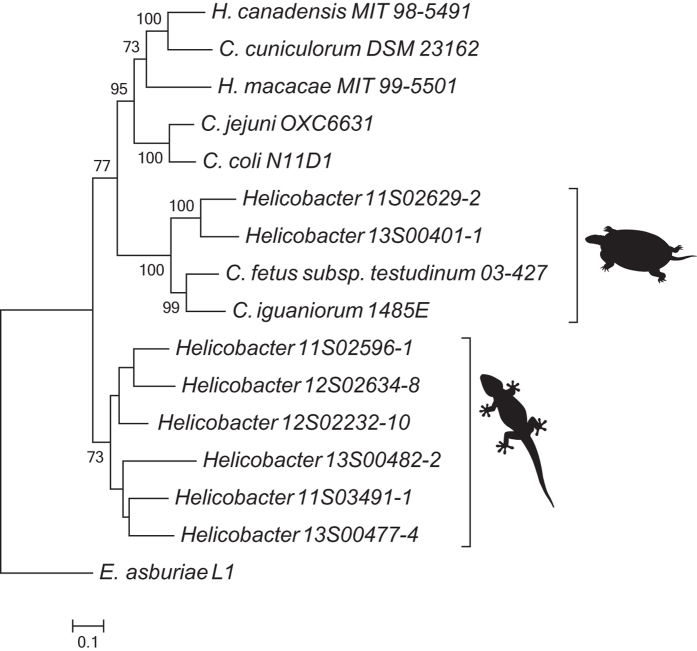
Rooted single gene maximum likelihood dendrogram based on *tcuC* from *Helicobacter* and *Campylobacter*. The chelonian- and squamate-associated strains are indicated with a chelonian or lizard, respectively. *Enterobacter asburiae* L1 was used as outgroup and root. Bootstrap values (≥70%) based on 500 repetitions are shown at the nodes of the dendrogram.

Initial screening showed that *tcuC* was conserved in all reptile-associated *Helicobacter* and *Campylobacter* lineages. This gene was used to elucidate the origin of *tcuC* and the *tcuRABC* locus. A maximum likelihood dendrogram based on *tcuC* from all *Helicobacter* and *Campylobacter* lineages shows that *tcuC* from the lizard-associated *Helicobacter* clade is divergent from all other lineages (Fig. 2). Interestingly, *tcuC* from the chelonian-associated *Helicobacter* lineages was most closely related to *tcuC* from the reptile-associated *Campylobacter* lineages.

## Discussion

*Helicobacter* phylogeny reflects host phylogeny only to a certain degree. In contrast to the 16S rRNA-based phylogeny, whole genome-based phylogeny shows that the reptile-associated *Helicobacter* lineages do not form one coherent clade. Instead, two separate and distantly related reptile-associated *Helicobacter* clades can be recognized; one associated with chelonians and one associated with squamates. Both clades are nested within the *Helicobacter* genus and do not form an apparent basal uniform sister clade to bird- and mammal-associated *Helicobacter* lineages. As *Helicobacter* phylogeny does not reflect amniotic vertebrate phylogeny, specific long-term coevolution of *Helicobacter* and its vertebrate host since the last common ancestor can be considered unlikely. Rather the whole genome-based phylogeny is suggestive of initial adaptation to a certain anatomical niche (e.g. gastric or intestinal), followed by a host jump and radiation in a particular host group (e.g. squamates or chelonians). This is most apparent in the squamate-associated *Helicobacter* clade, which is most closely related to the gastric mammal-associated *Helicobacter* clade, including *H*. *pylori*. The genes involved in urease production are conserved in all lineages and are indicative of a gastric niche. These genes show a similar phylogeny as the whole genome-based phylogeny, indicating long-term conservation within each lineage with an origin pre-dating the split between gastric mammal- and reptile-associated *Helicobacter* lineages. This is in support of an initial ancient adaptation to the gastric niche, followed by radiation in either mammals or squamates. Ancient host jumps between distantly related host-species have been reported previously for *Helicobacter* ^7^.

Interestingly, the phylogeny of reptile-associated *Helicobacter* parallels host association, i.e. each clade is confined to a phylogenetically distinct host type (either chelonian or squamate), indicating a high level of host specificity. In combination with the observed high diversity and deep branching within these clades, this supports long-term coevolution with, and extensive radiation within the respective reptilian host type.

Within the squamate-associated *Helicobacter* clade, *Helicobacter* strains obtained from recently wild-caught lizards from the same original geographic region clustered together, indicating an association with geographic origin. This association was not apparent amongst the other squamate-associated *Helicobacter* strains. As these were obtained from captive-held animals this signal could be obscured due to anthropogenic influences. Interspecies transmission in unnatural animal assemblies have been noted before for *Campylobacter* and *Helicobacter* ^2, 3^. Occurrence in multiple host types has been observed in reptile-associated *Helicobacter* as well, as an identical *Helicobacter* lineage was isolated from a captive-held lizard and a snake. However, the presence of *Helicobacter* in recently wild-caught reptiles, combined with the phylogenetic coherence, suggests that *Helicobacter* is likely a relevant constituent of the gastrointestinal microbiome in wild reptiles as well.

Growth at low temperature (≤25 °C) is observed in all reptile-associated *Helicobacter* lineages, but is not known from any *Helicobacter* species isolated from either birds or mammals. This shows that growth at low temperature is entirely associated with occurrence in a reptilian host. As this feature is observed in both distantly related chelonian- and squamate-associated *Helicobacter* clades, adaptation to the variable but on average low temperatures encountered in a poikilothermic reptilian host originated independently and at least twice in *Helicobacter* evolution. The observation that some squamate-associated *Helicobacter* lineages were able to grow at 42 °C and show the largest growth temperature range known for *Helicobacter* confirms the large thermal adaptation needed to survive in a poikilothermic host.

Genes putatively involved in tricarballylate catabolism (*tcuRABC*) were present in all reptile-associated *Helicobacter* lineages. Outside the reptile-associated *Helicobacter* lineages *tcuRABC* genes are only found in *H*. *canadensis* and *H*. *macacae*. In the closely related genus *Campylobacter* these genes are also predominantly found in the reptile-associated taxa *C*. *fetus* subsp. *testudinum* and *C*. *iguaniorum* ^8, 9^. This indicates that these genes may be important in survival in a reptilian host. As has been shown for *Salmonella enterica*, these genes potentially enable reptile-associated *Helicobacter* and *Campylobacter* lineages to use the citrate analog tricarballylate as carbon and energy source, which may provide an advantage for survival in a reptilian host^10^. Tricarballylate is toxic to ruminants by inhibiting aconitase and the citric acid cycle^11^. However, reptiles have been shown less susceptible to aconitase inhibition than mammals^12^. As such, reptiles are expected to be more tolerant to tricarballylate, which might be more abundant in the reptilian than in the mammalian gastrointestinal tract. Noteworthy, *tcuC* from chelonian-associated *Helicobacter* lineages is closer related to *tcuC* from reptile-associated *Campylobacter* lineages than to *tcuC* from lizard-associated *Helicobacter* lineages. This suggests lateral transfer of *tcuC* between *Helicobacter* and *Campylobacter*, potentially in a chelonian host, which shows the highest *Campylobacter* prevalence amongst reptiles^2^.

Based on our results, and in contrast to 16S rRNA-based phylogeny (Supplementary Figure S1), *Wolinella succinogenes* forms a clade together with *Helicobacter* species having unsheathed flagella. Excluding *W*. *succinogenes* from the *Helicobacter* phylogeny would leave it paraphyletic, which implies that *W*. *succinogenes* could be considered a member of the *Helicobacter* genus. Based on 16S rRNA, all reptile-associated *Helicobacter* lineages, both urease positive and negative, form a distinct clade together with *H*. *mustelae* and urease negative *H*. *pametensis*. As shown previously for *Helicobacter*, phylogenies based on 16S rRNA are discordant with 23S rRNA-based phylogenies and other data, which is consistent with the horizontal transfer of 16S rRNA gene fragments and loss of phylogenetic information^6^. As such, 16S rRNA might be less suitable for phylogenetic analysis of *Helicobacter*.

Based on the ANI values, all reptile-associated *Helicobacter* lineages included in this study represent novel species. With eight putative species, the diversity of *Helicobacter* in reptiles is high compared to the other vertebrate-associated Epsilonproteobacteria genera *Arcobacter* and *Campylobacter* (three and four species, respectively)^2^. An explanation of the high *Helicobacter* diversity could be a higher host or niche specificity, leading to more isolation, thereby facilitating diversification. Also, a more ancient introduction of *Helicobacter* in a reptilian host could have led to more extended diversification.

It has to be noted that several members of the *Helicobacter* genus are considered fastidious micro-organisms and likely many more *Helicobacter* lineages are present in reptiles than the ones included in this study, which may also include enterohepatic *Helicobacter* in squamates and gastric *Helicobacter* in chelonians. Furthermore, as all isolates were obtained from intestinal contents from cloacal swabs, the exact region of the gastrointestinal tract colonized by the reptile-associated *Helicobacter* isolates included in this study is not known, but rather the presumed anatomical niche is inferred from the position in the phylogenetic tree and the presence or absence of the urease locus. More culturing- and sequencing-based studies are needed to provide further insights in the exact diversity, phylogeny, and niche preference of *Helicobacter* in reptiles.

In conclusion, poikilothermic reptiles host a large diversity of *Helicobacter* lineages, which are distinct from bird- and mammal-associated *Helicobacter* species. These reptile-associated *Helicobacter* lineages provide novel insights in *Helicobacter* host adaptation, phylogeny, and evolution. Given the large diversity of *Helicobacter* in a limited number of well-investigated host species, it is expected that the total *Helicobacter* diversity in vertebrates far exceeds the currently known diversity. In all probability, further sampling of other reservoirs, preferably wild animals, should lead to an increase of *Helicobacter* diversity and a further refinement of *Helicobacter* phylogeny.

## Methods

### Strains

*Helicobacter* strains representing eight putative novel species were isolated from intestinal contents from cloacal swabs of chelonians and lizards as described previously^2^. All strains were isolated from captive-held animals from zoos, pet shops or private collections. None of the hosts had apparent intestinal illness or other clinical signs. By default, strains were grown on Columbia agar with 5% sheep blood (Oxoid, the Netherlands) in a microaerobic atmosphere (83.3 N_2_, 7.1% CO_2_, 3.6% H_2_, and 6% O_2_) at 37 °C for 48 h. To determine growth temperature range, the strains were also grown at 25 °C and 42 °C. Characteristics of all strains used in this study are summarized in Table 1.

### Whole genome sequencing

Sequencing of the reptile-associated *Helicobacter* strains was performed using Illumina MiSeq, 300 bp read length. The reads were assembled using SPAdes 3.1.1. The average coverage was 212× and average number of contigs was 59. The whole genome sequences of all reptile-associated *Helicobacter* strains have been deposited at GenBank. All available whole genome sequences of other (candidate) *Helicobacter* and *Wolinella* species were extracted from GenBank on March 9^th^ 2016. Genomic features and accession numbers of all *Helicobacter* and *Wolinella* genomes used in this study can be found in Supplementary Table S3.

In addition to this, the whole genome sequences of *Enterobacter asburiae* L1 (GenBank accession number CP007546.1), *Campylobacter coli* N11D1 (FBQY00000000.1), *C*. *cuniculorum* DSM 23162 (JHZL00000000.1), *C*. *fetus* subsp. *fetus* 82–40 (CP000487.1), *C*. *fetus* subsp. *testudinum* 03–427 (CP006833.1), *C*. *iguaniorum* 1485E (CP010995.1), and *C*. *jejuni* OXC6631 (CUVR00000000.1) were used in this study.

### Genome analysis

For prokaryote species delineation, the average nucleotide identity (ANI) can be used as an alternative for DNA-DNA hybridization (DDH)^13, 14^. A DDH species delineation of 70% corresponds to about 95% ANI^15^. Using the JSpecies ANI tool^16^, pair-wise ANI values based on the whole genome sequences were calculated for all strains used in this study.

To determine the presence of virulence factors, amino acid sequences of known *H*. *pylori* virulence factors (AlpA, AlpB, BabA, CagA, DupA, gGT, HopZ, IceA, IceA2, NapA, OipA, SabA, VacA) were aligned against the predicted amino acid sequences of each genome at an e-value cutoff of 1E-50 (1E-30 for IceA2) using BLAST.

### Orthologous grouping and phylogenomic reconstruction

An all versus all BLAST was performed for all predicted proteins of the whole genomes (Table 1) at an E-value cutoff of 1E-6. To determine the orthologous relationships of all proteins, the BLAST output was parsed by Orthagogue using default settings^17^. To determine the orthologous groups, Markov clustering (MCL) was performed using MCL-edge^18^. Genes encoding the proteins were aligned with each other within their respective orthologous groups using MUSCLE^19^. A super alignment of 603,413 nt was created by concatenating the aligned genes according to their position in *H*. *pylori* J99 if they were present in all isolates. Gaps were removed using TrimAl resulting in a 302,243 nt super alignment^20^. Based on this super alignment phylogenomic reconstruction and prediction of recombination events was performed using Gubbins^21^ with the default settings. Whole genome phylogeny was based on a gapless super alignment. Phylogenetic dendrograms were created using Fasttree^22^ or MEGA 6^23^.

For the 16S rRNA-based phylogeny, the 16S rDNA sequences were extracted from the genomes, aligned using MUSCLE^19^, gap positions and flanking sequences were removed resulting in a 1290 bp alignment. A phylogenetic tree was constructed using RaxML^24^ with a GTR model with gamma correction. The tree was rooted on *C*. *fetus* 82–40 and visualized using Figtree.

### Data availability

The data that support the findings of this study are included in this published article (and its Supplementary Information files) and are available in the GenBank database.

## Electronic supplementary material

Supplementary Figure S1

Supplementary Table S1

Supplementary Table S2

Supplementary Table S3

## References

[1] Schauer, D. B. In *Helic*obacter *p*ylori*: Physiology and genetics* (eds Mobley, H. L. T., Mendz, G. L. & Hazell, S. L.) (ASM Press, Washington (DC), 2001). 21290711

[2] Gilbert MJ (2014). Occurrence, diversity, and host association of intestinal *Campylobacter*, *Arcobacter*, and *Helicobacter* in reptiles. PLoS One.

[3] Schrenzel MD (2010). Genetic characterization and epidemiology of Helicobacters in non‐domestic animals. Helicobacter.

[4] Lawson, A. & Owen, R. *Helicobacter serpensis, a novel Helicobacter species isolated from snake faeces* (Zoonoses and public health Ser. 54, Blackwell Publishing, England, 2007).

[5] Stacy BA, Wellehan JF (2010). Fatal septicemia caused by *Helicobacter* infection in a pancake tortoise (*Malacochersus tornieri*). J. Vet. Diagn. Invest..

[6] Dewhirst FE (2005). Discordant 16S and 23S rRNA gene phylogenies for the genus *Helicobacter*: implications for phylogenetic inference and systematics. J. Bacteriol..

[7] Eppinger M (2006). Who ate whom? Adaptive *Helicobacter* genomic changes that accompanied a host jump from early humans to large felines. PLoS Genet.

[8] Gilbert MJ (2016). Comparative genomics of *Campylobacter fetus* from reptiles and mammals reveals divergent evolution in host-associated lineages. Genome Biol Evol.

[9] Gilbert MJ (2016). Comparative genomics of *Campylobacter iguaniorum* to unravel genetic regions associated with reptilian hosts. Genome Biol Evol.

[10] Lewis JA, Horswill AR, Schwem BE, Escalante-Semerena JC (2004). The tricarballylate utilization (*tcuRABC*) genes of *Salmonella enterica* serovar Typhimurium LT2. J. Bacteriol..

[11] Russell JB (1985). Enrichment and isolation of rumen bacteria that reduce *trans*-aconitic acid to tricarballylic acid. Appl. Environ. Microbiol..

[12] McIlroy J (1986). The sensitivity of Australian animals to 1080 poison. 9. Comparisons between the major groups of animals, and the potential danger nontarget species face from 1080 poisoning campaigns. Wildl. Res..

[13] Konstantinidis KT, Tiedje JM (2005). Genomic insights that advance the species definition for prokaryotes. Proc. Natl. Acad. Sci. USA.

[14] Konstantinidis KT, Ramette A, Tiedje JM (2006). The bacterial species definition in the genomic era. Philosophical Transactions of the Royal Society B: Biological Sciences.

[15] Goris J (2007). DNA-DNA hybridization values and their relationship to whole-genome sequence similarities. Int. J. Syst. Evol. Microbiol..

[16] Richter M, Rosselló-Móra R (2009). Shifting the genomic gold standard for the prokaryotic species definition. Proc. Natl. Acad. Sci. USA.

[17] Ekseth OK, Kuiper M, Mironov V (2014). orthAgogue: an agile tool for the rapid prediction of orthology relations. Bioinformatics.

[18] Enright AJ, Van Dongen S, Ouzounis CA (2002). An efficient algorithm for large-scale detection of protein families. Nucleic Acids Res..

[19] Edgar RC (2004). MUSCLE: multiple sequence alignment with high accuracy and high throughput. Nucleic Acids Res..

[20] Capella-Gutierrez S, Silla-Martinez JM, Gabaldon T (2009). trimAl: a tool for automated alignment trimming in large-scale phylogenetic analyses. Bioinformatics.

[21] Croucher, N. J. *et al*. Rapid phylogenetic analysis of large samples of recombinant bacterial whole genome sequences using Gubbins. *Nucleic Acids Res*. **43** (2014). 10.1093/nar/gku1196 PMC4330336 25414349

[22] Price MN, Dehal PS, Arkin AP (2009). FastTree: computing large minimum evolution trees with profiles instead of a distance matrix. Mol. Biol. Evol..

[23] Tamura K, Stecher G, Peterson D, Filipski A, Kumar S (2013). MEGA6: Molecular Evolutionary Genetics Analysis version 6.0. Mol. Biol. Evol..

[24] Stamatakis A (2014). RAxML version 8: a tool for phylogenetic analysis and post-analysis of large phylogenies. Bioinformatics.

[25] Haesebrouck F (2009). Gastric helicobacters in domestic animals and nonhuman primates and their significance for human health. Clin. Microbiol. Rev..

