# Supplementary material for: Whole genome-based phylogeny of reptile-associated Helicobacter indicates independent niche adaptation followed by diversification in a poikilothermic host
Source: Sci Rep. 2017 Aug 21;7:8387. doi: 10.1038/s41598-017-09091-7 (PMC5566214; doi:10.1038/s41598-017-09091-7)
Supplement: Supplementary file 1 — Supplementary Figure S1 [file 41598_2017_9091_MOESM1_ESM.pdf]

Whole genome-based phylogeny of reptile-associated *Helicobacter* indicates independent niche adaptation followed by diversification in a poikilothermic host

Maarten J. Gilbert, Birgitta Duim, Arjen J. Timmerman, Aldert L. Zomer, and Jaap A. Wagenaar

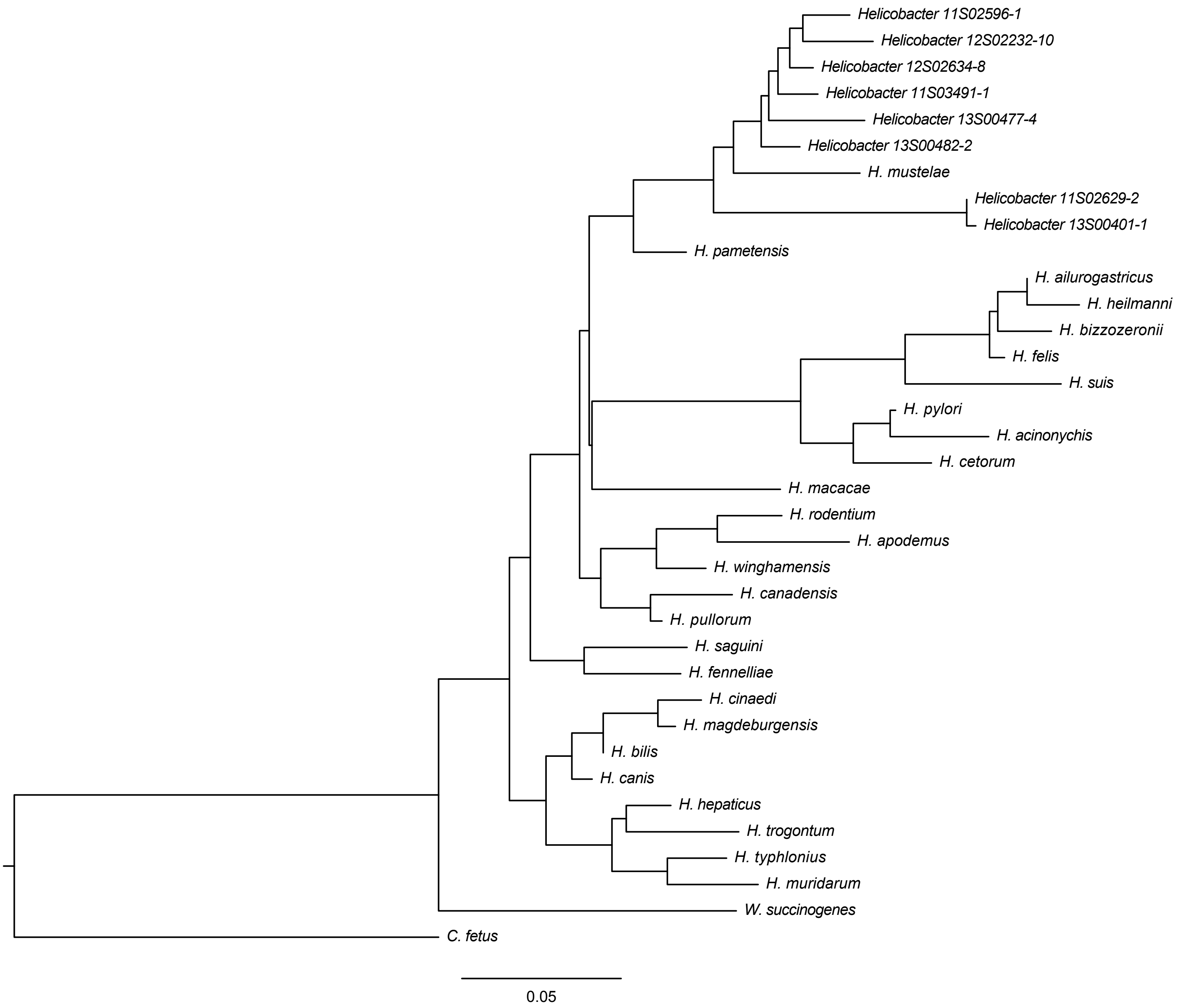

Supplementary Figure S1. Phylogenetic dendrogram based on 16S rRNA for all *Helicobacter* strains used in this study. *C. fetus* strain 82-40 was used as outgroup and root.
