# Supplementary material for: Whole genome-based phylogeny of reptile-associated Helicobacter indicates independent niche adaptation followed by diversification in a poikilothermic host
Source: Sci Rep. 2017 Aug 21;7:8387. doi: 10.1038/s41598-017-09091-7 (PMC5566214; doi:10.1038/s41598-017-09091-7)
Supplement: Supplementary file 2 — Supplementary Table S1 [file 41598_2017_9091_MOESM2_ESM.pdf]

Whole genome-based phylogeny of reptile-associated *Helicobacter* indicates independent niche adaptation followed by diversification in a poikilothermic host

Maarten J. Gilbert, Birgitta Duim, Arjen J. Timmerman, Aldert L. Zomer, and Jaap A. Wagenaar

Supplementary Table S1. Average nucleotide identity (ANI) values (%) based on BLAST for all *Helicobacter* strains used in this study. Reptile-associated *Helicobacter* strains are shown in bold.

Strain	**1**	**2**	**3**	**4**	**5**	**6**	7	8	9	10	11	12	13	14	15	16	17	18	19	20	21	22	23	24	25	26	27	**28**	**29**	30	31	32	33	34	35
**1 *Helicobacter* 11S02596-1**	100	72	77	74	72	71	66	66	66	66	66	66	66	66	67	67	67	67	67	67	68	67	68	66	67	66	66	66	66	66	66	66	66	66	66
**2 *Helicobacter* 11S03491-1**	72	100	75	71	74	73	67	66	66	65	66	65	65	66	67	67	67	67	67	67	67	66	67	67	67	67	66	66	66	67	67	67	67	66	65
**3 *Helicobacter* 12S02232-10**	77	75	100	74	75	73	67	66	66	65	66	65	65	66	66	67	67	67	67	67	67	66	67	67	67	66	66	66	66	67	67	66	67	66	65
**4 *Helicobacter* 12S02634-8**	74	71	74	100	72	70	67	67	66	66	66	66	66	66	67	67	67	67	67	67	68	67	67	66	67	66	66	66	66	66	66	66	66	66	66
**5 *Helicobacter* 13S00477-4**	72	74	75	71	100	72	67	66	66	65	65	65	64	66	66	66	66	67	67	66	67	66	67	67	67	67	66	66	66	67	67	67	67	66	65
**6 *Helicobacter* 13S00482-2**	71	74	73	70	72	100	67	66	66	65	65	65	65	66	66	67	67	67	67	67	67	66	67	67	67	66	66	67	67	67	67	67	67	66	65
7 *H. cetorum MIT 99-5656*	66	67	67	67	67	67	100	82	82	67	67	67	66	68	66	66	66	66	66	66	66	66	66	66	67	66	67	66	66	66	66	66	66	66	65
8 *H. acinonychis Sheeba*	67	66	67	67	66	66	82	100	89	67	67	67	67	67	66	65	66	66	66	66	66	66	66	66	66	66	66	65	65	66	66	65	66	65	65
9 *H. pylori J99*	66	66	66	67	66	66	82	89	100	67	67	67	67	68	66	65	65	66	66	66	66	66	66	65	65	65	66	65	64	66	66	65	66	65	64
10 *H. bizzozeronii CIII-1*	67	65	66	66	65	65	67	67	67	100	73	73	72	72	66	65	65	65	65	65	66	65	65	65	65	65	65	64	64	65	65	65	65	65	65
11 *H. felis ATCC 49179*	66	66	65	66	65	65	66	67	67	73	100	72	71	71	66	65	65	65	65	65	66	65	65	65	65	65	65	64	64	65	65	65	65	65	65
12 *H. ailurogastricus ASB7*	67	65	66	66	65	65	66	67	67	73	72	100	82	72	65	64	65	64	65	65	66	65	65	64	65	64	65	65	65	64	64	64	64	65	65
13 *H. heilmanii ASB1.4*	66	65	65	66	65	65	66	67	67	73	71	82	100	71	65	65	66	65	65	66	67	66	65	66	66	65	66	65	65	65	65	65	65	65	66
14 *H. suis HS1*	66	66	66	66	66	65	67	67	68	72	71	72	71	100	65	65	65	65	66	66	66	65	65	65	65	65	66	65	65	65	65	65	65	65	64
15 *H. mustelae 12198*	67	67	66	67	66	66	66	66	66	66	66	66	65	65	100	67	67	66	67	67	67	67	67	67	67	66	67	65	65	66	66	66	66	66	66
16 *H. pametensis ATCC 51478*	67	67	67	67	67	67	66	65	65	65	65	65	65	65	67	100	67	67	67	67	67	67	67	67	67	66	67	66	66	67	67	66	66	66	66
17 *H. typhlonius MIT 97-6810*	67	67	67	67	67	67	66	66	66	65	65	65	65	66	67	67	100	79	74	74	69	69	69	69	70	69	68	66	66	67	68	68	68	69	66
18 *H. hepaticus ATCC 51449*	67	68	67	67	68	67	66	65	65	65	65	64	64	65	67	67	79	100	75	75	68	68	68	69	69	68	67	66	66	68	68	69	68	69	65
19 *H. magdeburgensis MIT 96-1001*	67	67	67	67	67	67	66	66	66	65	65	65	65	65	67	67	75	75	100	94	69	71	70	71	76	68	68	66	66	68	68	68	69	68	66
20 *H. cinaedi ATCC BAA-847*	67	67	67	67	67	67	66	66	66	65	66	65	65	66	67	67	74	75	94	100	69	71	74	70	75	68	68	66	66	68	68	67	69	68	66
21 *H. macacae MIT 99-5501*	68	67	67	68	67	67	66	66	66	66	66	66	66	66	67	67	68	68	68	68	100	71	70	67	68	67	68	66	66	68	67	67	67	67	66
22 *H. canis NCTC 12740*	67	67	67	68	67	67	67	66	66	66	66	66	66	66	67	67	68	69	70	71	71	100	70	68	70	67	68	66	66	67	67	67	69	67	67
23 *H. fennelliae MRY12-0050*	68	67	67	67	67	67	66	66	66	65	65	65	65	65	66	67	69	68	69	73	71	70	100	68	69	67	67	66	66	67	67	67	67	67	65
24 *H. trogontum ATCC 700114*	66	67	67	66	67	67	66	66	66	65	66	65	65	65	67	67	68	69	70	70	67	68	69	100	83	71	69	67	67	68	68	68	68	67	65
25 *H. bilis ATCC 51630*	67	67	67	67	67	67	67	66	66	66	66	66	65	66	67	68	69	70	76	75	68	70	69	83	100	71	71	67	67	67	67	68	69	68	66
26 *H. muridarum ST1*	66	67	67	66	67	67	66	66	65	65	65	64	64	65	66	67	68	69	68	68	67	67	67	71	71	100	69	67	67	67	68	69	67	68	65
27 *H. saguini MIT 97-6194*	67	67	66	67	66	66	66	66	66	66	66	66	66	66	66	66	68	67	68	68	68	68	68	69	70	69	100	67	66	67	67	67	67	67	66
**28 *Helicobacter* 11S02629-2**	66	66	66	66	66	66	65	64	64	64	64	64	64	64	65	65	65	66	66	66	66	66	65	67	67	67	66	100	82	66	66	66	66	65	64
**29 *Helicobacter* 13S00401-1**	66	66	66	66	67	67	66	65	65	64	64	65	64	65	65	66	66	66	66	66	66	66	66	67	67	67	66	82	100	66	66	66	66	66	64
30 *H. pullorum MIT 98-5489*	66	67	67	66	67	67	66	66	65	64	64	64	64	65	66	66	67	68	67	67	67	67	67	67	67	67	66	66	66	100	78	73	72	72	66
31 *H. canadensis MIT 98-5491*	67	67	67	67	67	67	66	66	66	65	65	64	65	66	66	67	68	68	68	68	68	67	68	68	68	68	67	66	66	78	100	73	72	72	66
32 *H. apodemus MIT 03-7007*	66	66	67	66	67	67	66	65	65	64	64	64	64	65	66	66	68	68	67	67	67	66	67	67	67	68	66	66	65	73	73	100	72	72	65
33 *H. winghamensis ATCC BAA-430*	66	67	67	66	67	67	66	66	65	65	65	64	64	65	66	66	68	68	69	69	68	68	67	68	68	67	67	66	66	72	72	72	100	75	66
34 *H. rodentium ATCC 700285*	66	66	66	66	66	66	65	65	65	65	65	65	65	65	66	66	69	69	68	67	67	67	67	67	67	67	67	65	65	72	72	72	75	100	66
35 *W. succinogenes DSM 1740*	66	65	66	66	65	65	65	65	65	65	65	65	65	65	66	66	66	66	66	66	67	66	66	66	66	65	66	65	65	66	66	66	66	66	100
