# Supplementary material for: Whole genome-based phylogeny of reptile-associated Helicobacter indicates independent niche adaptation followed by diversification in a poikilothermic host
Source: Sci Rep. 2017 Aug 21;7:8387. doi: 10.1038/s41598-017-09091-7 (PMC5566214; doi:10.1038/s41598-017-09091-7)
Supplement: Supplementary file 4 — Supplementary Table S3 [file 41598_2017_9091_MOESM4_ESM.pdf]

Whole genome-based phylogeny of reptile-associated *Helicobacter* indicates independent niche adaptation followed by diversification in a poikilothermic host

Maarten J. Gilbert, Birgitta Duim, Arjen J. Timmerman, Aldert L. Zomer, and Jaap A. Wagenaar

Supplementary Table S3. Features of the genomes used in this study.

Strain	Size (bp)	Scaffolds	GC content (%)	CDS	rRNA*	tRNA	Virulence factors**	Accession number
Helicobacter 11S02596-1	1976285	50	41.4	1810	2	38	3	MLAM00000000
Helicobacter 11S02629-2	1884348	62	35.7	1672	1	36	2	MLAN00000000
Helicobacter 11S03491-1	1833365	34	33.8	1693	2	37	4	MLAO00000000
Helicobacter 12S02232-10	1906227	55	35.0	1900	1	39	4	MLAQ00000000
Helicobacter 12S02634-8	1895292	62	41.4	1823	2	37	3	MLAP00000000
Helicobacter 13S00401-1	1841089	60	34.8	1617	1	35	2	MLAR00000000
Helicobacter 13S00477-4	1781914	46	32.6	1711	2	37	4	MLAS00000000
Helicobacter 13S00482-2	1914003	109	33.5	1798	2	37	4	MLAT00000000
H. acinonychis Sheeba	1557588	2	38.2	1546	2	36	11	NC_008229.1
H. ailurogastricus ASB7	1578404	9	47.6	1514	1	37	4	CDMG01.1
H. apodemus MIT 03-7007	2114943	628	33.0	2033	3	37	2	JRPC01.1
H. bilis ATCC 51630	2474805	131	34.9	2285	4	40	3	JMKW01.1
H. bizzozeronii CIII-1	1807534	2	45.9	1870	3	37	4	NC_015674.1
H. canadensis MIT 98-5491	1623845	1	33.7	1575	4	41	1	NZ_CM000776.2
H. canis NCTC 12740	1924983	7	45.0	1792	3	41	4	AZJJ01.1
H. cetorum MIT 99-5656	1847790	2	35.5	1724	2	37	10	NC_017735.1
H. cinaedi ATCC BAA-847	2240130	1	38.3	2325	2	40	4	NC_020555.1
H. felis ATCC 49179	1672681	1	44.5	1668	3	37	3	NC_014810.2
H. fennelliae MRY12-0050	2155647	49	37.9	2122	1	38	3	BASD01.1
H. heilmanii ASB1.4	1804601	1	47.8	1812	2	42	5	HE984298.2
H. hepaticus ATCC 51449	1799146	1	35.9	1798	1	38	3	NC_004917.1
H. macacae MIT 99-5501	2358757	12	40.6	1941	3	37	2	AZJI01.1
H. magdeburgensis MIT 96-1001	2084456	360	38.8	2040	2	38	4	JRPE01.1
H. muridarum ST1	2353752	92	32.7	2118	2	46	2	JRPD01.1
H. mustelae 12198	1578097	1	42.5	1421	3	39	2	NC_013949.1
H. pametensis ATCC 51478	1433836	13	40.1	1366	4	39	1	JADE01
H. pullorum MIT 98-5489	1919070	131	34.2	1915	2	33	2	ABQU01.1
H. pylori J99	1643831	1	39.2	1504	2	36	12	NC_000921.1
H. rodentium ATCC 700285	1810652	29	37.0	1793	2	37	3	JHWC01.1
H. saguini MIT 97-6194	2922612	178	34.7	2651	2	38	3	JRMP01.1
H. suis HS1	1635292	136	39.9	1645	1	30	4	ADGY01.1
H. trogontum ATCC 700114	2767028	129	33.1	2550	3	38	3	JRPL01.1
H. typhlonius MIT 97-6810	1920832	1	38.9	1912	3	40	4	NZ_LN907858.1
H. winghamensis ATCC BAA-430	1654865	55	35.5	1654	2	34	2	ACDO01.1
W. succinogenes DSM 1740	2110355	1	48.5	2074	3	41	2	NC_005090.1

* draft genome assemblies. May be incomplete

** AlpA, AlpB, BabA, CagA, DupA, gGT, HopZ, IceA, IceA2, NapA, OipA, SabA, VacA
